# The effects of the ‘active before school’ programme on the academic skills of 8–9-year-old children: a physically and cognitively engaging intervention

**DOI:** 10.3389/fpubh.2024.1402901

**Published:** 2024-09-03

**Authors:** Agata Korcz, Jana Krzysztoszek, Łukasz Bojkowski, Agnieszka Koszałka-Silska, Maryna Khorkova, Anna Gomołysek, Michał Bronikowski

**Affiliations:** ^1^Department of Didactics of Physical Activity, Poznan University of Physical Education, Poznan, Poland; ^2^Department of Psychology, Poznan University of Physical Education, Poznan, Poland; ^3^Department of Pedagogy, Poznan University of Physical Education, Poznan, Poland

**Keywords:** early childhood education, before-school programme, technology, school skills, physical activity, intervention

## Abstract

**Introduction:**

Literature underscores the significance of exercise and cognitive stimulation for achieving academic success. This study aims to investigate the effects of the technology-based “Active Before the First School Bell” programme, comparing the effects of two school-based interventions (physical activity vs. cognitive engagement) on the academic skills of 8–9-year-old children.

**Methods:**

This encompasses their school skills, visual-motor coordination, levels and attitudes towards physical activity, and fitness. The study involved 88 primary school children (age: 8.3 years, 58.0% girls). To assess the programme’s distinct effects children were categorised into three groups. The first group (*n* = 31) participated in cognitive classes (CEG), the second (*n* = 27), in physical activity classes (PAEG), and the third (*n* = 30), was a control one (CG). A 12-week intervention, consisting of three 15-min sessions per week before school, was implemented. Self-report questionnaires gauged levels of physical activity. Academic skills were assessed using a battery of diagnostic methods for school failure in early childhood education. Fitness was measured using selected items from the Eurofit test battery. Pre- and post-test measures were collected and analysed, employing one-way ANOVA on ranks with Dunn’s post-hoc tests.

**Results:**

Significant post-test differences between the groups were observed in visual spatial function, with the PAEG outperforming the CG, and in auditory and language functions, where both experimental groups outperformed the CG. Results suggest that before school physical activities may be more effective than cognitive activities in improving the academic skills.

**Conclusion:**

The short-term effects obtained provide insights for implementing before-school programmes for children in the early school years.

## 1 Introduction

A plethora of studies and reports from many national and international organisations, including the WHO (World Health Organization), UNICEF (United Nations Children’s Fund), OECD (the Organization for Economic Co-operation and Development), and the EU (European Union) offer comprehensive insights into child health and well-being in Europe. The understanding of children’s needs has sharpened, emphasising the need for personalised care (1) and raising awareness of the pivotal role children play as both current individuals and future adults.

There is a lack of up-to-date, adequate, and reliable data on some crucial aspects of children’s health and well-being. These aspects are often studied separately, lacking a holistic approach that considers children and families, along with the broader contextual influencing their health trajectories (2). Significant knowledge has been amassed about childhood diseases, well-organised within the Classification of Diseases. Progress has also been made in understanding child development, prenatal care, nutrition, hygiene, and family strengths (3). Yet, there is a paucity of information concerning cognitive and social–emotional development, social cohesion, and even comparable knowledge of eating habits and physical activity (PA) (4).

The start of primary school in Poland, typically around ages 6–7 years, is an important period for children’s physical and cognitive development. This stage, known as the stage of concrete operations, according to Piaget (5), is characterised by the development of logical thinking skills and problem-solving abilities based on personal experiences. It is also when children develop memory and cooperative skills. At this age, children begin to grasp temporal concepts like past, present, and future, contributing to planning actions to achieve their goals. Furthermore, they can analyse complex concepts, such as addition and subtraction, and formulate cause-and-effect relationships (6). Cognitive development during this period affects emotional, social functions, and, in general, personal development. Piaget (5) considered that motor and cognitive skills are closely related, a connection empirically validated by studies (7). Applied science pursues initiatives to explore these relationships and address the challenges of children’s health and well-being. These initiatives often take the form of school programmes and interventions, integrating thematic areas and interactions within the educational environment. Schools provide cost-effective settings for health education programmes and are critical for developing the health-related knowledge of children and adolescents (8). A review of 56 high-quality reviews of school-based interventions (9) with various occupations implemented in school settings, and concerning all focus areas found some positive effects. Significant effects were observed in interventions aimed at increasing PA (10). Moreover, physical-activity-promoting interventions, including school-based promotion and those designed to improve fitness levels have reported positive effectiveness results in 6 of 9 trials (11). The remaining intervention programmes produced insignificant effects confirming their effectiveness. Yuksel et al. (12) also found that physical activity-oriented programmes tend to yield higher success rates in all variables. School-based interventions hold significant potential for promoting PA and fitness. The abovementioned study highlights that the quality, duration, and prioritisation of PA interventions in comprehensive school-based programmes, as well as teacher capacity, are critical factors (12). Children benefit from early interventions to develop healthy behaviours and lifestyles throughout their lives (13). As early as 2000, the International Union for Health Promotion and Education (IUHPE) found that interventions were most effective when they focused on academic and social outcomes in addition to behaviour change (14).

One type of school intervention is before-school physical activity (PA) programmes, engaging students in PA before the regular school day. These programmes can contribute to meeting recommended children’s PA guidelines. PA has been linked to improved cognitive function, including attention, memory, and problem-solving skills. While the direct impact of before-school PA on academic performance is not fully established, some studies suggest that enhanced physical health and cognitive function may potentially contribute to better academic outcomes over time (15). PA can help reduce stress and anxiety, which positively influences a student’s ability to focus and perform well academically (16). Physically fit students tend to have better attendance rates and fewer health-related absences, leading to more consistent engagement with their studies. Furthermore, schools that prioritise PA opportunities by incorporating physical education (PE) classes, recess breaks, and active learning strategies may create a more holistic learning environment that benefits academic outcomes (17).

A systematic review by Donnelly et al. (18) demonstrated the growing popularity of programmes and interventions *n* = 137 (*n* = 64 cognitive function and *n* = 73 academic achievement studies meeting inclusion criteria) on the effects of PA, fitness, PE, and sports participation on cognition, learning, brain function and structure, academic achievement, and attention. The findings suggest positive associations between PA, fitness, cognition, and academic achievement, indicating a positive impact of PA on cognition, brain structure, and function. However, these findings are inconsistent, and the effects of numerous elements of PA on cognition, such as type, amount, frequency, and timing remain to be explored. In the aforementioned review (18), 32 studies analyzed the effects of acute PA attacks on academic performance or concentration/attention, including: 10 cross-sectional comparisons of academic performance among students with different levels of PA, eight studies of the effects of a single acute PA attack on tests of academic achievement, attention and concentration, and 14 studies of academic performance after implementation of PA interventions. The results were mixed (18): among the cross-sectional studies, four studies showed a positive association, three studies showed a positive association in some academic areas but not in others, two studies showed no association, and one study showed a negative association. In contrast, among the 14 studies that analyzed the PA intervention (18): five studies showed clear improvement, three studies showed improvement in some aspects of academic achievement or some students but not others, and six studies showed no improvement in academic achievement after PA. It is important to note that the relationship between PA and academic outcomes is complex and can be influenced by various factors, including the amount and intensity of PA, the quality of physical education programmes, and individual student characteristics. There is a need to better understand the synergies between education and children’s health during this period of development. More research is therefore required to determine mechanisms, long-term effects, and strategies for implementing interventions within the school environment (18). Participating in PA before school may potentially enhance children’s ability to concentrate and focus on academic tasks throughout the day. It is reported in this regards that PA promotes brain health by increasing blood flow and oxygen delivery to the brain, thereby improving its capacity to process information and support learning (19). Notably, interventions scheduled during the school day have shown mixed effectiveness due to increased academic demands and crowded curricula (20). As a result, before-school activities provide opportunities outside of school hours and are typically shorter than after-school programmes. This article examines the role of before-school activities in supporting student development, explores optimisation strategies, and discusses trends and challenges in this area for future research.

Despite the above findings, previous studies have predominantly focused on either physical or cognitive interventions in isolation, rather than directly comparing their effects. The specific rationale for this study stems from the need to understand the individual effects of physical and cognitive activities on children’s development when these activities are delivered separately. In addition, the timing of the interventions—delivered before the start of the school day—adds a unique dimension to the research, as this period may be particularly influential on pupils’ readiness and performance in subsequent academic activities. The study evaluated the impact of the “Active Before the First School Bell” programme, engaging 8–9-year-old children physically or cognitively, on academic skills, eye-hand coordination, attitudes towards PA, levels of PA, and physical fitness. The assumption is that the intervention programme, when combined with cognitive activities, would lead to significantly greater gains in academic skills compared to the same programme paired with PA. The intervention emphasising PA was, however, expected to result in positive changes in PA levels, attitudes towards PA, visual-motor coordination, and fitness-related parameters. Therefore, to meet the aims of the study, the effectiveness of two technology-based interventions was compared: (1) the physically active group with a video exercise programme, and (2) the cognitively active group with a cognitive stimulation game. By addressing these specific research needs, this study aims to provide empirical evidence that can inform educational and public health policies aimed at improving child well-being through targeted, pre-school interventions.

## 2 Methods

### 2.1 Study design and participants

This study was conducted during the winter of 2023, presenting process data from the “Active Before the First School Bell” programme. It involved 88 students (37 boys and 51 girls) aged 8–9 years (8.3 ± 0.5) from the second grade of four urban primary schools of city of Poznan. Each intervention (physical and cognitive engagement) was administered within the same school (organised by classes—one intervention type per class), with the exception of the control school (which was a school without intervention). The study protocol was approved by the Local Bioethics Committee (decision number 864/22) and conformed to the principles of the Declaration of Helsinki. Informed consent was obtained from the principals of the selected schools. Before data collection, we informed prospective students and parents of the purpose of the study, obtained written informed consent from the parents of all students, and obtained consent from the children. Parents and students were informed that the students’ answers would be confidential and would only be used for research purposes. Participation in the study was voluntary and students could withdraw at any time.

**Figure 1:**
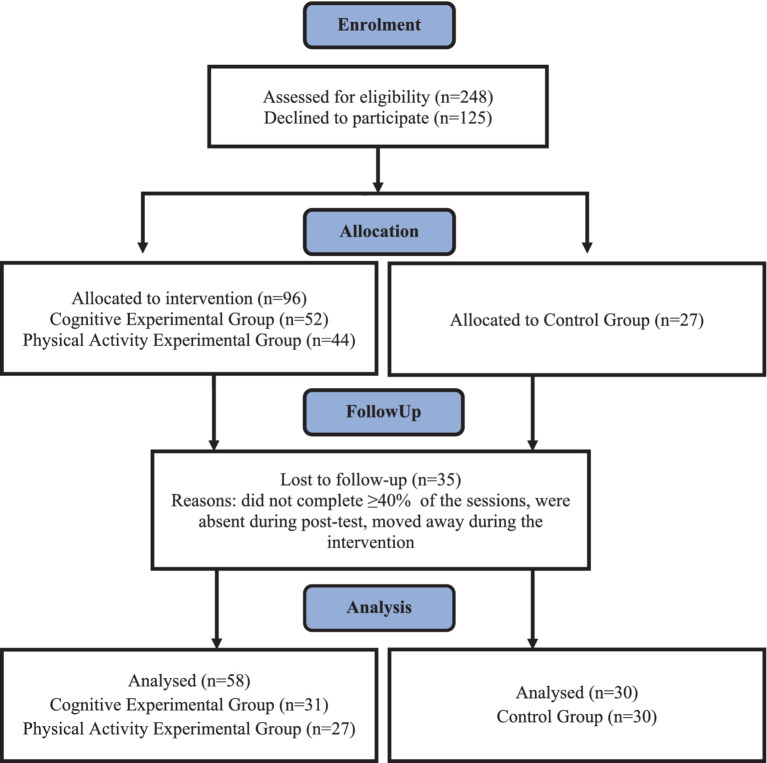
Flow chart of study participants.

The inclusion criterion for the study was that the students did not attend physical education (PE) classes with significantly more hours (physical education) than the standard number of PE hours per week (number of physical education hours according to the curriculum was 3 per week). Students were randomly allocated (by class) to three groups: (1) a cognitive experimental group (CEG), (2) a PA experimental group (PAEG), and (3) a control group (CG). The sample size was estimated using the GPower 3.1 software, with effect size = 0.25, alpha value = 0.05, power = 0.80, number of groups = 3, number of measurements = 2, the total sample size calculated was 105 for the present study. However, the number of students was lower than expected for two main reasons. The sessions started before school hours, which required extra efforts from the students and their families. In addition, financial constraints prevented the study group from being extended to another school. From 123 pupils interviewed (pre-test measurements) in the first term of the study, approximately 28.5% of students had incomplete data from both terms. This data loss was due to illness, non-participation in the fitness tests due to injuries, and dropout during programme. Only complete data sets from both study dates (1st date—February 2023, 2nd date—June 2023) and the results of students who had at least ≥40% participation in programme activities were used for statistical analysis. Ultimately, 31 students (13 boys and 18 girls) participated in the cognitive experimental group, 27 students (10 boys and 17 girls) in the PA experimental group, and 30 students (14 boys and 16 girls) in the control group. Figure 1 illustrates the flow of participants across the intervention study.

### 2.2 Intervention

Within the programme, the experimental groups participated in structured physical or cognitive intervention. Teachers (*n* = 6, three female and three male, including four physical education teachers and two early childhood education teachers) participated in the experiment and received training on its implementation and the use of the specified tools. These teachers were informed about the study’s objectives but were kept blind to the specific hypotheses. Regular face-to-face meetings or phone calls were conducted to support the teachers during the intervention programme. The fidelity of the programme’s implementation was systematically checked by the primary researcher through designated platforms (with login access for programme teachers and supervisors). As part of the research activity, the teachers conducted a 12-week intervention (March–May 2023). This intervention consisted of 15-min PA or cognitive classes, three times a week, just before the start of scheduled lessons (36 units in total). The 12-week duration was chosen to align with the school term, without holiday interruption, to control for confounding holiday-related effects.

The PA experimental group utilised the HOPSports Brain Breaks® Physical Activity Solutions platform (21), BB for short, which provided a video exercise programme. These exercises are web-based structured PA breaks designed to improve an individual’s health and education. The programme used only movement-based videos to teach movement skills and improve fitness, presented through animations and real instructors, with all students performing the same task simultaneously. The videos featured a range of fundamental movements, encompassing warm-up exercises, elements drawn from various sports and traditional dances, and traditional or popular music from different countries worldwide. Videos were selected by PE teachers. Each session comprised three to four videos. The selected videos were created in sets to be used for 15 min each. The activities took place in a classroom equipped with a projector, screen, speakers, and a computer.

Students in the cognitive experimental group utilised the CogniFit platform of cognitive stimulation games (22). This tool customised the training programme based on game-level progression to match each participant’s abilities, using an algorithm fed by an initial assessment of cognitive function. The training programme employs diverse games (over 50 games) incorporating visual, auditory, and cross-modal stimuli to train a broad spectrum of cognitive processes. Each game is used to train more than one cognitive ability, as they are relatively complex games in terms of cognitive demand. Also, each session comprises three games (tasks) to be used for 15 min on in the CogniFit personalised programme. It is crucial that participants complete the training sessions as designed. If participants exit the session before finishing all activities, they will be prompted to return and complete the remaining tasks to ensure progression. This system feature aims to maintain continuity in training and optimise the effectiveness of the intervention. Training took place in computer rooms designed for the training programme, with each workstation equipped with headphones. Permission has been obtained from HOPSports and CogniFit to use their platforms. The control group did not participate in the pre-school classes and followed the existing curriculum. The participants from that group were informed that they would have two assessments (at baseline and after 12 weeks), while they went about their daily activities in the meantime. In addition, it was mainly the parents who knew that their children had been allocated to the control group.

### 2.3 Measures

Participants underwent two assessment sessions, encompassing height and weight measurements, alongside self-reported PA. Trained staff collected data on body mass and height using anthropological instruments (Wunder Sa. Bi. Srl., Italy). Body mass index (BMI) was calculated by dividing body weight by the square of height in metres (kg/m^2^). Students received instructions on testing techniques, with each test item accompanied by specific instructions provided to the students. All measurements and tests occurred on school premises, utilising gymnasiums and classrooms. The questionnaires were administered individually to the children, with one-to-one assistance from a trained staff member, in conditions that ensured student comfort.

#### 2.3.1 Self-reported PA

PA was assessed using self-report items previously used in the Health Behaviour in School-aged Children (HBSC) study at national, regional, and international levels (23, 24).

Levels of moderate-to-vigorous PA (MVPA) were assessed using a Physical Activity Screening Measure (25). Participants responded to two questions: (1) In the past 7 days, on how many days did you get at least 60 min of total PA per day? and (2) In a typical or usual week, on how many days do you get at least 60 min of total PA per day? Response categories ranged from 0 day to 7 days (with 8 response categories). The MVPA index was calculated using the formula: MVPA = (Q1 + Q2)/2 where: MVPA = PA ratio; Q1 is the number of physically active days in the last 7 days; Q2 is the number of physically active days in a typical week. According to Prochaska et al. (25), this measure is reliable (ICC = 0.77). This measure was also previously employed in Poland for a similar age group (26).

#### 2.3.2 Behavioural measures of self-reported PA

The Attitude towards Physical Activity Scale (APAS) was used to measure children’s attitudes and perceptions towards various aspects of engagement in PA. The APAS questionnaire comprises eight sections, including a demographic section (covering gender, age, grade, height, and weight), of which five sections were used for this study. These sections corresponded to five scales, namely: (1) Benefits: a 10-item scale constructed to measure students’ perceived benefits of PA; (2) Importance: a 5-item scale constructed to measure students’ perceived importance of PA; (3) Fun: a 14-item scale constructed to measure students’ interest in doing PA; (4) Fitness: an 8-item scale designed to measure students’ confidence in their own fitness; (5) Personal best: a 5-item scale designed to measure students’ orientation to their personal best goals when engaging in PA. Responses to the items were collected using a 4-point Likert scale ranging from 1 (‘strongly disagree’), 2 (‘disagree’), 3 (‘agree’) to 4 (‘strongly agree’). The original English version of the questionnaire had been validated in an earlier study (27) using Rasch analysis, which provided empirical support for the scales; reliability, one-dimensionality, response category effectiveness, and the absence of gender-related item functioning differentials (DIF). Cronbach’s alpha values calculated on the present total sample for the entire scale and each subscale in the pretest were 0.89, and in the posttest were 0.92 (with 0.67 and 0.83 for ‘benefits’, 0.38 and 0.60 for ‘importance’, 0.73 and 0.80 for ‘fun’, 0.69 and 0.67 for ‘fitness’, 0.70 and 0.68 for ‘personal best’). The APAS questionnaire has been used in several previous studies and in a similar age group (28).

#### 2.3.3 Battery of methods for diagnosing school failure causes in pupils aged 7–9 (Battery-7/9)

The Battery-7/9, as outlined by Bogdanowicz et al. (29), is designed for the psychological and educational diagnosis of specific reading and writing difficulties in children aged 7–9 years. It covers the diagnosis of auditory and language functions (awareness and phonological skills), visual–spatial functions (visual processing), perceptual-motor integration, and the speed of naming visual stimuli. Tasks that required timing were measured using stopwatches. All tests from this battery were administered separately to the children, where they were tested on a one-to-one basis. In our study, only selected tests from Battery 7/9 were used:

##### 2.3.3.1 Figure compare test (visual–spatial functions)

The purpose of this test is to assess pattern and spatial perception, visual speed, and visual attention. The test involves seven patterns (geometric figures) that the respondent must assess. The participant is presented with a model figure, followed by six patterns below it, from which they must select the shape corresponding to the one shown above. The test results are scored based on the accuracy of the responses and the total time taken to complete them. The index’s minimum value is 0, and the maximum is 7. Additionally, there is an auxiliary index that is dependent on the participant’s speed. The coefficient alpha for the Polish version is 0.41.

##### 2.3.3.2 Quick naming test (visual–auditory integration)

The purpose of this test is to assess visual–auditory integration, long-term verbal memory, and rapid automatic word recall. A set of three rapid naming tasks has been developed to assess naming speed. In each task, the subject is required to quickly name specific items. These tasks involve: naming 50 coloured stars (black, blue, green, red, yellow), naming 40 simple drawings (cheese, ice cream, sun, ship, clock, banana, etc.), and naming 29 drawings and 19 letters mixed pseudo-randomly. The measure in this test is the time taken to name all the objects. The time is recorded separately for each task. Any errors made by the respondent are added to the time index, with each error considered as an additional second. The coefficient alpha for the Polish version is 0.56 for colours, 0.74 for pictures, and 0.75 for colours and pictures together.

##### 2.3.3.3 Auditory–visual integration test (perceptual-motor-integration)

The purpose of this test is to evaluate the ability to integrate multimodal and motor information, transfer information from one modality to another, and assess memory. During the test, the examiner dictates successive series of sounds, and the participant writes them down on a piece of paper. The primary metric for analysis is the number of correct answers, with a range from a minimum of 0 to a maximum of 12. The coefficient alpha for the Polish version is 0.42.

##### 2.3.3.4 Selected auditory-linguistic test—phonological memory test (auditory and linguistic functions)

This test is designed to assess the phonological aspect of auditory-linguistic functions and auditory memory for words. The moderator reads a series of words to the respondent and asks the respondent to recall the series from memory. The primary metric for analysis is the number of correct answers, with a range from a minimum of 0 and a maximum of 18. The coefficient alpha was not calculated.

### 2.4 Assessment of physical fitness

The health-related physical fitness status of the participants was assessed using the European Test of Physical Fitness (30). Four physical fitness indicators were used: (1) Plate Tapping (PLT, speed of limb movement), (2) Sit-and-Reach (SAR, flexibility), (3) Sit-Ups (SUP, abdominal muscular endurance), (4) 10 × 5 m Shuttle Run (SHR, running speed, agility). All tests, except for the sit-ups and shuttle run, were performed twice and the best performance was recorded.

### 2.5 Assessment of visual-motor coordination (reaction time)

The Piórkowski apparatus (APK) is a test of visual-motor coordination (reaction time). In this test, the subject’s objective is to respond to a flashing light stimulus by pressing the right button as quickly as possible, ensuring the response occurs during the exposure of the light (by pressing one of the 10 buttons, each one corresponding to one of 10 related lights). The APK device was placed on a table with appropriate height to provide comfortable operation using both hands in a standing position. Correct response and incorrect response were recorded. The parameters in the study comprised a 60-s test with a stimulus presentation frequency of 30 pulses within that time frame. This test has already been used in the study by Merkisz et al. (31).

### 2.6 Data analysis

The Statistical Package for Social Science (SPSS) was used for all analyses. The distribution of normality was calculated using the Kolmogorov–Smirnov and Shapiro–Wilk tests for all continuous variables. Therefore, due to the non-normality of the data, they were expressed as medians and interquartile range (according to the formula: q3–q1) quartiles (1 and 3). To compare variables before (pre) and after (post) the 12-week programme, the Wilcoxon test was used. To compare pre- and post-change separately between the three groups (PAEG, CEG, and CG), the one-way ANOVA on ranks (Kruskal–Wallis H-test) with Dunn’s post-hoc (non-parametric) was applied. In all cases, a value of *p* < 0.05 was considered statistically significant.

## 3 Results

At the pre-test stage, one-way ANOVA on ranks revealed significant group differences and main effects across the three groups (time), as summarised in Table 1. Specifically, before the start of the programme (pre-test), statistically significant differences between the study groups were found in the following areas: visual–spatial functions (pts) (*p* = 0.02),; auditory–visual integration—colours (sec) (*p* = 0.01), visual-motor coordination—reaction time (no) (*p* = 0.01), MVPA (*p* = 0.04), two variables from the APAS scale: Fun—interest in doing PA (0.02) and Personal Best—trying to achieve personal best (*p* < 0.01).

**Table 1:** Results of the one-way ANOVA on ranks analysis between groups (CEG, PAEG, CG) in pre- and post-tests.

Variables	*p*-value for differences between groups (CEG, PAEG, CG)	
	Pre-test	Post-test
School skills—selected features		
Visual–spatial functions (pts)	**0.02**	**0.03**
Visual–spatial functions (sec)	0.53	0.89
Visual–auditory integration—colours (sec)	**0.01**	0.05
Visual–auditory integration—drawings (sec)	0.15	0.28
Visual–auditory integration—drawings/letters (sec)	0.48	0.23
Visual–auditory integration (s ∑ total)	0.08	0.18
Perceptual-motor integration (pts)	0.42	0.41
Auditory and linguistic functions (pts)	0.76	**< 0.01**
**Visual-motor coordination—reaction time (no)**	**0.01**	0.68
**MVPA (number of days/week)**	**0.03**	0.12
Selected physical fitness tests		
PLT (s)	0.46	0.58
SAR (cm)	0.07	0.06
SUP (no)	0.16	0.29
SHR (s)	0.18	**<0.01**
The attitude towards PA		
Benefits: perceived benefits of PA (pts)	0.25	0.10
Importance: importance of PA (pts)	0.07	0.11
Fun: interest in doing PA (pts)	**0.02**	0.48
Fitness: self-confidence on physical fitness (pts)	0.44	0.45
Personal best: trying to do personal best (pts)	**<0.01**	**0.04**

*p* < 0.05. CEG, cognitive experimental group; PAEG, physical activity experimental group; CG, control group; no, number; s, seconds; pt, points; cm, centimeters; ∑, total; MVPA, moderate to vigorous PA; PLT, disc tapping (a test of hand speed); SAR, supine sit-ups (a test of flexibility); SUP, supine sit-ups (a measure of core strength—abdominal muscular endurance), SHR, 10 × 5 m shuttle run (a test of running speed, agility).

Post-hoc for pre-test comparisons: Visual–spatial functions (pts): PAEG vs. CG: *p* = 0.02; Auditory–visual integration—colours (sec): PAEG vs. CEG: *p* = 0.02; Visual-motor coordination—reaction time (no): CEG vs. CG: *p* = 0.01; Fun—interest in doing: PAEG vs. CG *p* = 0.02; Personal best—trying to do personal best: CEG vs. PAEG: *p* = 0.00.

Post-hoc test for post-test comparisons: Visual–spatial functions (pts): PAEG vs. CG: *p* = 0.03; Auditory and linguistic functions (pts): CEG vs. CG: *p* = 0.00 and PAEG vs. CG: *p* = 0.00; SHR (s): PAEG vs. CG: *p* = 0.00 and PAEG vs. CEG: *p* = 0.03; Personal best—trying to do personal best: PAEG vs. CG: *p* = 0.05. Bold values are *p*-value < 0.05.

To test the main hypotheses of the study, the three groups were compared between pre-test and post-test. For school skills, one-way ANOVA on ranks revealed a significant time by group interaction effect for visual–spatial functions (*p* = 0.03), auditory and language functions (*p* < 0.01). For physical fitness, one-way ANOVA on ranks showed a significant time by group interaction effect only for the SHR (*p* < 0.01). and one variable from the APAS scale: Personal Best—trying to achieve personal best (0.04).

Children’s pre- and post-measurements across three groups for selected school skills, reaction time, PA, selected physical fitness tests, and all APAS variables are presented in Table 2. A comparison of the results shows a greater number of beneficial changes in CEG (nine statistically significant changes) compared to PAEG (seven statistically significant changes) and CG (five statistically significant changes). Statistically significant positive changes were found in both experimental groups concerning functions that have an impact on children’s school skills. In CEG, these changes occurred in four variables (out of eight): auditory–visual integration—pictures (*p* = 0.01), pictures/letters (*p* < 0.01) and total score (*p* < 0.01); and changes in auditory-linguistic functions (*p* < 0.01). In PAEG, three statistically significant changes were observed in the abovementioned abilities (out of eight), namely in the two variables of auditory–visual integration (pictures/letters *p* < 0.01, total score *p* < 0.01) and in perceptual-motor integration (*p* = 0.02). In CG, two changes (out of eight) were observed in the children’s school skills, specifically in visual–auditory integration—drawings/letters (*p* = 0.03) and auditory–visual integration in the total score (*p* = 0.01).

**Table 2:** Medians and interquartile ranges for study variables at pre- and post-tests for the intervention and control groups (*N* = 88).

Variables	CEG (*n* = 31)		*p*	PAEG (*n* = 27)		*p*	CG (*n* = 30)		*p*
	Pre-test	Post-test		Pre-test	Post-test		Pre-test	Post-test	
	Me (IQR)			Me (IQR)			Me (IQR)		
School skills—selected features									
Visual–spatial functions (pkt)	5.00 (2.00)	5.00 (2.00)	0.21	6.00 (1.00)	6.00 (2.00)	0.44	4.00 (3.00)	5.00 (3.00)	0.55
Visual–spatial functions (sec)	65.00 (23.80)	59.32 (18.51)	0.41	66.00 (25.00)	67.03 (20.70)	0.41	59.00 (26.00)	60.40 (29.50)	0.66
Visual–auditory integration—colours (sec)	53.65 (15.00)	49.10 (15.99)	0.06	46.00 (10.00)	41.96 (12.00)	0.05	49.00 (13.00)	47.00 (17.41)	0.19
Visual–auditory integration—drawings (sec)	45.00 (16.00)	41.80 (10.15)	**0.01**	41.00 (12.00)	39.80 (14.00)	0.09	42.09 (17.43)	41.26 (15.50)	0.70
Visual–auditory integration—drawings/letters (sec)	52.00 (22.00)	47.50 (16.90)	**0.01**	51.00 (12.54)	44.06 (14.40)	**<0.01**	50.78 (17.70)	48.66 (16.21)	**0.03**
Visual–auditory integration (sec ∑ total)	149.00 (47.26)	139.48 (35.40)	**<0.01**	136.00 (37.04)	130.51 (32.56)	**<0.01**	137.00 (48.00)	131.86 (40.50)	**0.01**
Perceptual-motor integration (pts)	6.00 (2.00)	6.00 (2.00)	0.27	6.00 (2.00)	7.00 (2.00)	**0.02**	6.00 (1.00)	6.00 (3.00)	0.06
Auditory and linguistic functions (pts)	6.00 (3.00)	8.00 (3.00)	**<0.01**	7.00 (4.00)	7.00 (3.00)	0.17	7.00 (4.00)	6.00 (2.50)	0.26
**Visual-motor coordination—reaction time (no)**	23.50 (8.00)	27.00 (3.0)	**<0.01**	24.00 (6.00)	28. (3.00)	**<0.01**	27.00 (5.50)	28.0 (3.00)	**0.01**
**MVPA (number of days/week)**	3.50 (3.00)	5.25 (2.50)	**<0.01**	5.00 (3.00)	5.75 (3.00)	0.23	3.50 (2.50)	4.00 (1.50)	**0.01**
Selected physical fitness tests									
PLT (s)	16.00 (4.00)	15.47 (2.48)	**<0.01**	17.44 (3.00)	16.42 (3.38)	0.11	16.80 (2.71)	16.50 (2.54)	0.08
SAR (cm)	5.00 (12.50)	2.00 (8.00)	0.25	9.00 (8.00)	7.00 (7.50)	**0.01**	3.25 (9.50)	4.0 (6.00)	0.77
SUP (no)	17.00 (5.00)	19.0 (5.00)	**<0.01**	17.00 (5.00)	18.00 (6.00)	**0.02**	15.00 (5.50)	18.00 (5.00)	**<0.01**
SHR (s)	24.00 (3.00)	22.97 (2.05)	**0.02**	24.17 (1.13)	24.50 (2.26)	0.97	24.89 (2.07)	26.15 (2.84)	0.06
The attitude towards physical activity									
Benefits: perceived benefits of PA (pts)	34.00 (6.00)	34.50 (9.00)	0.84	34.50 (6.00)	35.50 (5.00)	0.06	33.00 (5.50)	34.00 (5.00)	0.49
Importance: importance of PA (pts)	19.00 (3.00)	19.00 (3.00)	0.38	20.00 (1.00)	20.00 (2.00)	0.44	19.00 (3.00)	19.00 (3.00)	0.74
Fun: interest in doing PA (pts)	50.00 (8.00)	50.00 (7.00)	0.78	52.50 (3.00)	51.00 (4.00)	0.34	48.00 (8.50)	50.00 (6.00)	0.20
Fitness: self-confidence on physical fitness (pts)	29.00 (5.00)	28.00 (6.00)	0.48	29.00 (3.00)	29.00 (3.00)	0.46	28.50 (4.00)	28.00 (5.00)	0.93
Personal best: trying to do personal best (pts)	19.00 (3.00)	19.50 (3.00)	0.17	20.00 (0.00)	20.00 (1.00)	0.92	19.00 (2.00)	19.00 (2.00)	0.35

*p* < 0.05; CEG, cognitive experimental group; PAEG, physical activity experimental group; CG, control group; Me, median; IQR, interquartile ranges; no, number; s, seconds; pt, points; cm, centimeters; ∑, total; MVPA, moderate to vigorous physical activity; PLT, disc tapping (a test of hand speed); SAR, supine sit-ups (a test of flexibility); SUP, supine sit-ups (a measure of core strength—abdominal muscular endurance); SHR, 10 × 5 m shuttle run (a test of running speed, agility). Bold values are *p*-value < 0.05.

Regarding physical fitness, a statistically significant change in the test of running speed (10 × 5 m shuttle run) was observed only in the CEG (*p* = 0.02). Statistically significant changes in the abdominal muscular endurance test (sit-ups from a lying position) were observed in all groups, including CEG and CG (*p* < 0.01), PAEG (*p* = 0.02). An unfavourable change in the PAEG was observed in the flexibility test (*p* = 0.01) (supine torso in a sit-up). Among the statistically significant changes observed in CEG and CG, there were changes in MVPA (CEG *p* < 0.01and CG *p* = 0.01), as well as in the reaction time for eye-hand coordination (*p* = 0.00 for CEG and PAEG and *p* = 0.01 for CG). In CEG (*p* < 0.01) a statistically significant change was found in the hand movement speed test (tapping the pucks). In attitudes towards PA, no statistically significant changes were observed, neither in the pre-test nor in the post-test.

## 4 Discussion

This study aimed to assess the impact of two interventions on the school skills of 8–9-year-old children. One intervention emphasised cognitive activities, while the other focused on PA, each with specific expected outcomes. This is important as improvements in physical health and cognitive functioning can lead to enhanced academic performance (15). Modern educational institutions play a crucial role in shaping positive attitudes towards education, physical activity (PA), and health among students. It is vital to recognise the long-term consequences of decisions made during the early school years.

The study revealed that the ‘Active Before the First School Bell’ programme had a significant impact. The physical activity engagement group (PAEG) outperformed the control group (CG) in visual–spatial function. Both groups outperformed the control group (CG) in auditory and language functions. Additionally, PAEG and CEG, compared to CG, excelled in striving for personal in sports activities and selected the physical fitness component (SHR—a test of running speed, agility). These results align with the findings of Shore et al. (16), Kulp and Zhu (32) and Bruijn et al. (33).

Shore et al. (16) observed an indirect effect of PA on attention and knowledge acquisition. Bruijn et al. (33) compared a 14-week aerobic and cognitively engaging intervention, revealing that more MVPA led to improved mathematics and spelling performance in both intervention groups. Xu et al. (34) obtained similar results with a 16-week PA programme of 15 min per day before school. At the same time, an analysis by Martínez-López et al. (35) found that all short-term high-intensity sessions improve children’s cognitive performance. García-Hermoso et al. (36) discovered significant changes in language and mathematics performance for students (8–10 years old) involved in a before school 8 weeks PA programme (delivered daily for 30 min). Kulp and Zhu (32) found similar results with a 10-week pre-school exercise programme of 45 min one morning per week, where a typical session consisted of a dynamic warm-up, cardiorespiratory fitness activity, muscular fitness activity, group sports or tag gamer, and a cool-down stretch. In this experiment children benefited from participating in the programme by improving their reading test performance. Incidentally, it is worth noting that different results in this regard were obtained by Van den Berg et al. (37), who evaluated the effect of a 9-week programme of daily 10-min active breaks of moderate to vigorous intensity, which did not significantly improve cognitive performance in children aged 9–12 years.

In contrast, when the results of all three study groups were compared separately before and after the intervention, CEG exhibited nine significant and objectively improved test results. PAEG demonstrated seven such improvements, and CG, five. Notably, all three groups showed enhanced performance in the test of visual–auditory integration (overall score) and visual–auditory integration (drawing/ letters). CEG also showed improvements in specific tests related to this integration and in auditory-linguistic functions (details of the test tasks are outlined in the methodological section). In the test of perceptual-motor integration, PAEG displayed a progressive improvement, which also positively impacted the level of perceptual-motor integration. The changes in the CG can be attributed to the general developmental trajectory observed in children of this age. Despite the lack of intervention, the children were involved in the school curriculum, which included physical education activities. These stimuli contribute, to varying degrees, to developmental changes and may have been reflected in the research findings.

The results from the cited research intervention indicate that following a 12-week programme, both experimental groups (PAEG and CEG) showed positive changes in certain functions related to children’s school skills. To explain why the CEG displayed more positive changes in school skills than the PAEG or CG, it is noteworthy that cognitive training, designed to directly affect these skills, may have been more focused on developing specific school-related abilities such as critical thinking, problem-solving, or attention skills than the physical training, in which the PAEG participated to a greater extent in terms of time spent on the activity or involvement in it.

These findings are consistent with those of other researchers who have conducted international interventions focusing on cognitive functions and have described various benefits for students in terms of their academic performance. For example, Conesa and Duñabeitia (38) studied the effects of a computer-based training programme (CogniFit) on students’ executive functions and academic performance, revealing improvements in the experimental group compared to the control in areas like working memory and academic performance. Similar conclusions were reached by Reina-Reina et al. (39), who found that an 8-week intervention using CogniFit led to improved reading comprehension.

Regarding the results obtained in the selected motor tests, it is evident that all groups have experienced an improvement in reaction time skills, i.e., eye-hand coordination, supine sit-ups (a measure of core strength). Only CEG group obtained better results in a test of running speed, agility (SHR), whereas in the PAEG no better results were obtained in this respect, which may be due to the nature of the activity proposed, which may have insufficiently stimulated the development of a specific motor skill.

Comparing our results with international research, it is worth mentioning the intervention carried out by Mok et al. (28) in which students performed a series of brief exercises based on short videos twice during the school day in the classroom. This intervention led to a positive shift in attitudes towards PA, with improvement seen in six of the seven variables listed in the APAS scale among children aged 8–11 years. Similar results were also found by Glapa et al. (40), who studied the attitudes of Polish students aged 9–11 years.

This study exhibits several strengths and limitations. Notably, the intervention was designed to be practical for future implementation in schools, and the fact that it was conducted entirely by school staff is a notable strength. This approach offers a realistic perspective on the intervention’s effectiveness on a larger scale. The survey instruments used were mostly suitable for 8–9-year-olds, but the need for interviewer assistance in this age group is recognised as a crucial and strong element of the study.

Yet, there are limitations to consider. One instrument, the Pupils’ Attitudes to Physical Activity Questionnaire (APAS), proved to be challenging for respondents to understand, which introduces a limitation to the study’s findings. Additionally, technical difficulties in extracting data from the physical activity monitors (number of steps) resulted in insufficient data for analysis. Consequently, PA was solely assessed using self-reported questions about the children’s PA levels. The accuracy of children’s responses may be questionable. Furthermore, the data were collected in schools in the city of Poznan, which limits the generalisability of the results to the broader Polish child population. Additionally, we did not analyse gender differences due to the small sample sizes in each group. Another limitation is the absence of a second post-test to assess the results in the long term. The timing of the programme, conducted during the winter and spring, may have influenced the PA levels of the study participants, and should be considered when interpreting the results. Furthermore, in the case of the Battery-7/9 instrument, the same student was retested (post-tested) after 3 months, which may have led to a better knowledge of the tests and thus to better results.

The findings of this study have practical implications for schools. Implementing structured physical or cognitive activities before the school day can enhance various aspects of child development. Schools should consider adopting programmes like the “Active Before the First School Bell” programme, tailoring activities to their specific goals. Future research should investigate the combined effects of physical and cognitive activities to determine whether an integrated approach yields greater benefits. Long-term studies are needed to assess the sustained impact of such interventions. Additionally, examining the optimal duration and intensity of these activities, as well as their effects on diverse student populations (different age groups, considering how the sexes differ), would be valuable. Understanding the underlying mechanisms could inform the design of more effective school-based programmes.

## 5 Conclusion

This study shows that school-based interventions that combine PA and cognitive/computer-tailored interventions appear promising for improving academic skills in primary school children. We conclude that schools can implement feasible pre-school activities. Such programmes do not take time away from academics and can be administered directly by school staff. An excellent value of the proposed programme is the relative simplicity of the tools (platforms) used as well as easiness of the implementation. Future research should be based on further exploring the effects of implementing this programme of both combined and isolated cognitive and PA activities on children’s school skills, PA levels and physical fitness.

## Data Availability

The raw data supporting the conclusions of this article will be made available by the authors, without undue reservation.

## References

[1] Montgomery-Taylor S Klaber B Watson M. Developing personalised outcome measures (POMs) in a child health population. Int J Integr Care. (2017) 17:340. doi: 10.5334/ijic.3658

[2] Tyack Z. The greatest challenges and solutions to improve children’s health and well-being worldwide in the next decade and beyond: using complex systems and implementation science approaches. Front Pediatr. (2023) 11:1128642. doi: 10.3389/fped.2023.1128642, PMID: 36923277 PMC10009164

[3] Mukherjee T. Child well-being at the crossroads: the impact of parental work and lifestyle choices from a socio-ecological perspective In: Deb S, editor. Child safety, welfare and well-being (2022). (Singapore: Springer). 143–62. doi: 10.1007/978-981-16-9820-0_9

[4] Laurent CWS Burkart S Andre C Spencer RM. Physical activity, fitness, school readiness, and cognition in early childhood: a systematic review. J Phys Act Health. (2021) 18:1004–13. doi: 10.1123/jpah.2020-0844 34140418 PMC9297301

[5] Piaget J. The theory of stages in cognitive development In: Green D Ford MP Flamer GB, editors. Measurement and piaget. New York, NY: McGraw-Hill. 1–11.

[6] Petrova B. Characteristics of cognitive functioning in children of primary school age. Pedagogical Forum. (2021) 9:28–37. doi: 10.15547/PF.2021.004

[7] Van der Fels IMJ Wierike S Hartman E Elferink-Gemser M Smit J Visscher C. The relationship between motor skills and cognitive skills in 4–16-year-old typically developing children: a systematic review. J Sci Med Sport. (2015) 18:697–703. doi: 10.1016/j.jsams.2014.09.007, PMID: 25311901

[8] Nagy-Pénzes G Vincze F Bíró É. A school intervention's impact on adolescents' health-related knowledge and behavior. Public Health Front. (2022) 10:822155. doi: 10.3389/fpubh.2022.822155 PMC8963932 35359760

[9] Suto M Miyazaki C Yanagawa Y Takehara K Kato T Gai R. Overview of evidence concerning school-based interventions for improving the health of school-aged children and adolescents. J Sch Health. (2021) 91:499–517. doi: 10.1111/josh.13021, PMID: 33818772

[10] Naude CE Visser ME Nguyen KA Durao S Schoonees A. Effects of total fat intake on bodyweight in children. Cochrane Database Syst Rev. (2018) 2:CD012960. doi: 10.1002/14651858.CD012960 29446437 PMC6491333

[11] MacArthur G Caldwell DM Redmore J. Individual-, family-, and school-level interventions targeting multiple risk behaviours in young people. Cochrane Database Syst Rev. (2018) 10:CD009927. doi: 10.1002/14651858.CD009927.pub2, PMID: 30288738 PMC6517301

[12] Yuksel HS Şahin FN Maksimovic N Drid P Bianco A. School-based intervention programs for preventing obesity and promoting physical activity and fitness: systematic review. Int J Environ Res Public Health. (2020) 17:347. doi: 10.3390/ijerph17010347 31947891 PMC6981629

[13] Blümel M Achstetter M Hengel P Schwarzbach M Busse R. 4.K. Workshop: promoting and enhancing health literacy through school interventions. Eur J Pub Health. (2022) 32:ckac129.246. doi: 10.1093/eurpub/ckac129.246

[14] International Union for Health Promotion and Education (IUHPE). The evidence of health promotion effectiveness: Shaping public health in a new Europe. Brussels – Luxembourg. Paris, France: Jouve Composition & Impression. (2000).

[15] Woodforde J Alsop T Salmon J. Effects of school-based before-school physical activity programs on children’s physical activity levels, health and learning-related outcomes: a systematic review. Br J Sports Med. (2022) 56:740–54. doi: 10.1136/bjsports-2021-104470 34815223

[16] Shore E Cheung PC Hyde E Gazmararian JA. Physical activity opportunities and academic outcomes of fourth grade elementary school students in Georgia. J Sch Health. (2020) 90:25–31. doi: 10.1111/josh.12846, PMID: 31770813

[17] Healthy People. Physical activity: enhanced school-based physical education. (2020). Available at: https://www.healthypeople.gov/2020/tools-resources/evidence-based-resource/behavioral-social-approaches-increase-physical-activity (Accessed August 17, 2023).

[18] Donnelly J Hillman C Castelli D Etnier J Lee S Tomporowski P. Physical activity, fitness, cognitive function, and academic achievement in children. A systematic review. Med Sci Sports Exerc. (2016) 48:1197–222. doi: 10.1249/MSS.0000000000000901, PMID: 27182986 PMC4874515

[19] Bartee RT Heelan KA Dority BL. Longitudinal evaluation of aerobic fitness and academic achievement among schoolchildren. J Sch Health. (2018) 88:644–50. doi: 10.1111/josh.12666 30133778

[20] Dobbins M Husson H DeCorby K LaRocca RL. School-based physical activity programs for promoting physical activity and fitness in children and adolescents aged 6 to 18. Cochrane Database Syst Rev. (2013) 2013:CD007651. doi: 10.1002/14651858.CD007651.pub2, PMID: 23450577 PMC7197501

[21] HOPSports. Brain breaks® physical activity solutions platform. Available at: http://hopsports.com/what-is-brainbreaks (Accessed November 16, 2023).

[22] CogniFit. Platform of cognitive stimulation games. Available at: https://www.cognifit.com (Accessed November 16, 2023).

[23] Currie C Zanotti C Morgan A Currie D De Looze M Roberts C. Social determinants of health and well-being among young people. Health behaviour in school-aged children (HBSC) study. International report from the 2009/2010 survey In: Health policy for children and adolescents, vol. 6. Copenhagen, Denmark: WHO Regional Office for Europe (2012). Available at: https://iris.who.int/handle/10665/326406

[24] Mazur J Dzielska A Małkowska-Szkutnik A. Zdrowie i zachowania zdrowotne uczniów 17-letnich na tle zmian w drugiej dekadzie życia [Health and health behaviors of 17-year-old students against the background of changes in the second decade of life]. Instytut Matki i Dziecka: Warszawa (2020) (in Polish). (Accessed November 16, 2023).

[25] Prochaska JJ Sallis JF Long BA. Physical activity screening measure for use with adolescents in primary care. Arch Pediatr Adolesc Med. (2001) 155:554–99. doi: 10.1001/archpedi.155.5.554 11343497

[26] Bronikowski M Bronikowska M Pluta B Maciaszek J Tomczak M Glapa A. Positive impact on physical activity and health behaviour changes of a 15-week family focused intervention program: “juniors for seniors”. Biomed Res Int. (2016) 2016:1–8. doi: 10.1155/2016/5489348, PMID: 27766262 PMC5059515

[27] Mok MM Chin MK Chen S Emeljanovas A Mieziene B Bronikowski M. Psychometric properties of the attitudes toward physical activity scale: a Rasch analysis based on data from five locations. J Appl Meas. (2015) 16:379–400. 26771567

[28] Mok MM Chin MK Korcz A Popeska B Edginton CR Uzunoz FS. Brain breaks® physical activity solutions in the classroom and on attitudes toward physical activity: a randomized controlled trial among primary students from eight countries. Int J Environ Res Public Health. (2020) 17:1666. doi: 10.3390/ijerph17051666, PMID: 32143392 PMC7084371

[29] Bogdanowicz M Sajewicz-Radtke U Radtke BM Kalka D. Bateria metod diagnozy przyczyn niepowodzeń szkolnych u uczniów w wieku 7-9 lat. Bateria-7/9 [a battery of methods for diagnosing the causes of school failure in students aged 7–9. Battery-7/9] In: Pracownia Testów Psychologicznych i Pedagogicznych. Gdańsk, Poland (2015) (in Polish)

[30] EUROFIT. Handbook for the EUROFIT tests of physical fitness. 2nd ed. Strasbourg: Publishing and Documentation Service Strasbourg, Sports Division Council of Europe (1993).

[31] Merkisz J Galant M Karpiński D Orszulak B. Wykorzystanie Aparatu Piórkowskiego do oceny porównawczej zdolności psychomotorycznych kierowcy przed i po treningu symulatorowym [Using the Camera Piórkowskiego to comparatively evaluate thepsychomotor ability of a driver before and after simulation training]. Logistyka. (2014) 4:826–30. (in Polish)

[32] Kulp AJ Zhu X. Before school exercise effects on fitness and academic performance in schoolchildren: a retrospective case-controlled study. J Teach Phys Educ. (2021) 41:738–43. doi: 10.1123/jtpe.2021-0058

[33] De Bruijn AG Kostons DD Van Der Fels IM Visscher C Oosterlaan J Hartman E. Effects of aerobic and cognitively-engaging physical activity on academic skills: a cluster randomized controlled trial. J Sports Sci. (2020) 38:1806–17. doi: 10.1080/02640414.2020.1756680, PMID: 32567975

[34] Xu T Byker EJ Gonzales MR. Ready to learn: the impact of the morning blast physical activity intervention on elementary school students. Mov Health Exerc. (2017) 6:1–12. doi: 10.15282/mohe.v6i1.137

[35] Martínez-López EJ Ruiz-Ariza A De La Torre-Cruz M Suárez-Manzano S. Alternatives of physical activity within school times and effects on cognition. A systematic review and educational practical guide. Psicología Educativa Revista de los Psicólogos de la Educación. (2021) 27:37–50. doi: 10.5093/psed2020a16

[36] García-Hermoso A Hormazábal-Aguayo I Fernández-Vergara O González-Calderón N Russell-Guzmán J Vicencio-Rojas F. A before-school physical activity intervention to improve cognitive parameters in children: the active-start study. Scand J Med Sci Sports. (2020) 30:108–16. doi: 10.1111/sms.13537, PMID: 31410887

[37] Van den Berg V Saliasi E de Groo RH Chinapaw MJ Singh AS. Improving cognitive performance of 9–12 years old children: just dance? A randomized controlled trial. Front Psychol. (2019) 10:174. doi: 10.3389/fpsyg.2019.00174, PMID: 30787899 PMC6372522

[38] Conesa PJ Duñabeitia JA. Effects of computer-based training on children’s executive functions and academic achievement. J Educ Res. (2021) 114:562–71. doi: 10.1080/00220671.2021.1998881

[39] Reina-Reina C Conesa PJ Duñabeitia JA. Impact of a cognitive stimulation program on the reading comprehension of children in primary education. Front Psychol. (2022) 13:985790. doi: 10.3389/fpsyg.2022.985790, PMID: 36687904 PMC9853897

[40] Glapa A Grzesiak J Laudanska-Krzeminska I Chin MK Edginton CR Mok M. The impact of brain breaks classroom-based physical activities on attitudes toward physical activity in polish school children in third to fifth grade. Int J Environ Res Public Health. (2018) 15:368. doi: 10.3390/ijerph15020368, PMID: 29466285 PMC5858437

